# Thermographic‐Infrared Changes During Surgical Osteotomy Influenced by the Motor Type, System, and Diameter

**DOI:** 10.1155/ijod/9220381

**Published:** 2026-01-16

**Authors:** Daniel Alvitez-Temoche, Franco Mauricio, Berly Delgado-Cumpa, Julia Medina, Fran Espinoza-Carhuancho, Ivan Calderon, Frank Mayta-Tovalino

**Affiliations:** ^1^ Academic Department, Vice-Rectorate for Research, Faculty of Dentistry, Universidad Nacional Federico Villarreal, Lima, Peru, unfv.edu.pe; ^2^ Academic Department, Bibliometrics Evidence Evaluation and Systematic Reviews Group (BEERS) Human Medicine Career, Universidad Científica del Sur, Lima, Peru, cientifica.edu.pe; ^3^ Evidentia Research Group, Universidad Nacional Mayor de San Marcos, Lima, Peru, unmsm.edu.pe; ^4^ Vicerrectorado de Investigación, Universidad San Ignacio de Loyola, Lima, Peru, usil.edu.pe

**Keywords:** dental implant, implant system, motor type, thermographic-infrared changes

## Abstract

**Background:**

Thermographic‐infrared changes during osteotomy surgery depend on the type of motor used, size, and system of the dental implant. This study undertakes the evaluation of these thermal variations for successful optimization of dependent surgical procedures while aiming toward reduced thermal damage to the bone tissue.

**Methods:**

An in vitro comparative study was conducted to assess the thermographic‐infrared changes during surgical osteotomy influenced by the motor type, diameter, and dental implant system. The Checklist for Reporting in vitro Studies (CRIS) guidelines were adopted to report the findings. Bones with fractures, structural damage, or anomalies were omitted. The bones were stored at −20°C, then thawed, cleaned, disinfected, and calibrated. The implant motors (Coxo, W&H, Dentflex, and Baby Driller) were calibrated for equal performance. Standardized drilling procedures were followed using initial, pilot, second, and final drills. A Fluke TiS55+ thermographic camera recorded temperature changes every 10 s, being maintained at 30 cm.

**Results:**

ANOVA analysis revealed significant differences in temperature variations between the implant systems and drill types (*p* = 0.001). The Coxo and Dentflex motors showed higher mean temperatures (26.57 and 27.65°C, respectively) compared to the W&H and Baby Driller (24.45 and 24.65°C, respectively). Regression analysis indicated that baseline (*β* = −0.67, *p*  < 0.01), pilot drill (*β* = 0.37, *p*  < 0.01), and second drill temperatures (*β* = 0.80, *p*  < 0.01) significantly influenced the final drilling temperature. However, the implant system (*p* = 0.39) was not a significant predictor of the final temperature.

**Conclusion:**

The motor type and implant system have a major effect on the thermal changes that occur during surgical osteotomies. Clinicians should administer motor types and implant systems to minimize thermal damage to the bone; thus, the performance in dental implant surgeries can be improved. Therein, we have patient safety and longevity of dental implants.

## 1. Introduction

Tooth loss is frequently associated with oral pathologies such as caries or periodontal disease and can also be caused by trauma or accident [1]. To illustrate the magnitude of this problem, more than half of adult’s report at least one missing permanent tooth, according to the American Association of Oral and Maxillofacial Surgeons (AAOMS) [2]. Factors such as the number of missing teeth, bone absorption, and periodontal status are determinants for adequate treatment, such as dental implants, which completely replace the tooth loss, improving both masticatory function and the patient’s emotional state and proving to be a more advantageous option compared to other treatments [2–4].

For the placement of an implant, a perforation is made in the bone, inserting the implant in the same surgical site, called the osteotomy area [4]. Over time, a physiological process called osseointegration occurs, in which the surrounding bone structure heals and is deposited on the implant, ensuring successful anchorage between the two elements [4, 5]. However, there are certain requirements for this natural mechanism to occur, ensuring the health of the participating bone cells [5]. According to the literature, the main risk factor is thermal damage, which prevents bone regeneration and increases osteoclastic activity, leading to necrosis. Research postulates that the temperature limit ranges between 44 and 47°C for up to 1 min; if the temperature exceeds 60°C, permanent damage to the cells occurs [4, 6, 7]. In addition, the surgical technique employed, excessive postoperative inflammation, the aseptic and antisepsis protocol during the intervention, or an allergic response are considered as elements that prevent successful osseointegration [6, 8].

With regard to thermal regulation in the treatment, there are several determinants for the temperature increase, such as the model and diameter of the drill, the deterioration of the drill, the application of the coolant, and the drilling technique, among others [5, 7]. Currently, technology can be used to guide and plan the surgery in the most optimal way, such as computer‐assisted implant surgery (CAIS) or static CAIS, minimizing invasiveness during treatment, improving accuracy when placing the implant, and reducing anchorage complications or postsurgical repercussions [9, 10].

For reasons of implant research, it is not possible to study implants in the oral cavity of a living human being; therefore, different substitutes that simulate the jaws are used, such as bovine bone (usually ribs) or cuts made from polyurethane foam, certified by the American Society for Testing and Materials [6, 11]. In this same area of research, there are a large number of studies that evaluate thermal changes with infrared thermography, yielding more precise results in the procedure. Through the images, the exact temperature of the element to be evaluated is observed [4]. Infrared thermography can be used in various studies as it is a noninvasive, remote, and nonionizing procedure, for example, when evaluating temporomandibular disorders or analyzing the biomechanical load [12, 13].

Therefore, the aim of this study was to evaluate infrared‐thermographic changes during surgical osteotomy as influenced by the type of motor, diameter, and dental implant system in an in vitro study.

## 2. Material and Methods

### 2.1. Study Design

An in vitro comparative study was conducted to evaluate the thermographic‐infrared changes during surgical osteotomy as influenced by the motor type, diameter, and dental implant system. The Checklist for Reporting in vitro Studies (CRIS) guidelines were used to report the findings of this study [14].

### 2.2. Sample Size

**Figure 1:**
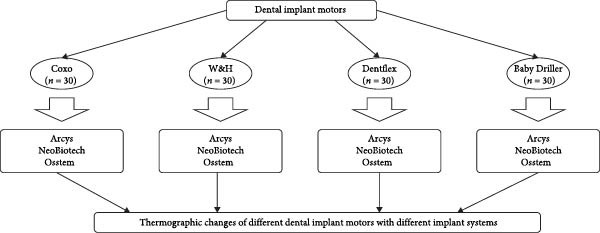
Diagram of the group distribution.

Sample size was computed by the formula for comparing means in Stata 17.0 software with an alpha significance level of 0.05 and a statistical power (beta) of 0.80. The calculation yielded a sum of specimens (cow rib bones) divided into 4 groups of *n* = 30 specimens each one (Figure 1).

### 2.3. Selection Criteria

The materials selected for the study were cow rib bones acquired from the Yerbateros farmyard in Lima, Peru. Healthy ribs were chosen for uniformity in bone size and quality: no fractures and bone density type 2, according to the Mish classification. Those rib bones with evident fractures or damage, bone density more than type 2, the Mish classification, evidence of disease and bone abnormalities, as well as those that did not comply with the size uniformity determined for the study, were excluded from the study.

### 2.4. Cow Bone Preparation

After this procedure, the bone sample sections were held at a constant −20°C until needed to prevent structural degradation and maintain physical integrity. The bone requiring surgery was subsequently frozen at room temperature for 24 h to assure conducive thawing conditions and prevent cracks or latter fractures due to dramatic temperature changes. Each bone segment was cleaned thoroughly with normal saline to remove any surface debris. Antisepsis was accomplished with the topical application of a 10% povidone‐iodine solution to effectively remove any potential microbiological contamination. The segments were washed thoroughly again in sterile saline and then allowed to air dry in a segregated environment to avoid possible recontamination.

### 2.5. Motor Calibration

The implant motors (Coxo, W&H, Dentflex, and Baby Driller) were adjusted according to the manufacturers’ specifications to provide optimal performance. Rotational speed and torque verification were performed with a special test stand, adjusting for foot control responses and checking for stability with a load. The repeatability and precision of each motor were determined from the results of several milling cycles under controlled conditions. The tested motors showed consistent seated speed and applied torque. Therefore, these motors are ready for use in the simulated surgical procedures in the study (Table 1).

**Table 1:** Implant motors.

Company	Brand	City	Country	Technical specifications	Key differentiators
Dentflex	Dforce 1000	Sao Paulo	Brazil	Power supply 95–245 V, consumption power 400 VA, frequency 50–60 Hz	Intermittent operation mode‐1 min on/9 min off
Driller	600 baby brushless	Sao Paulo	Brazil	Power supply 127–220 V, consumption power 150 VA, frequency 50–60 Hz	Intermittent operation mode‐15 min working/15 min resting
Coxo	C‐Sailor	Guangdong	China	Power supply 230 V, consumption power 130 VA, frequency 50–60 Hz	Intermittent operation mode‐2 min on/10 min off
W&H	Implantmed plus	Bürmoos	Austria	200–40,000 rpm, torque 80 Ncm, ISO short micromotor, color touchscreen	Intuitive user interface, safe torque control, measurable implant stability, wireless control pedal, machining function for threading in bone

### 2.6. Surgical Milling

The milling of the surgical implant was performed according to specific procedures laid down by each of the dental implant systems (Arcys, NeoBiotech, and Osstem). A standardized sequence of drills was provided to achieve proper preparation of the implant bed. All drilling started correctly with the baseline drill to create and step the starting point and direct the trajectory, according to the implant manufacturer’s changed rotational speed and torque specifications. The pilot drill intended to deepen the initial hole while establishing the definite direction of the implant, while the control parameters (the rotational speed and torque) were steady to ensure accuracy and avoid deviation. The second drill was used to enlarge the pilot drill hole diameter, which is crucial to ensuring that the implant bed is adequately prepared for the initial stability of the implant. Finally, the third drill profiled the proper diameter and depth according to the use of the final implant, and the speed and torque were gently modified to prevent heat‐related damage to the surrounding bone.

### 2.7. Thermographic Measurement

**Figure 2:**
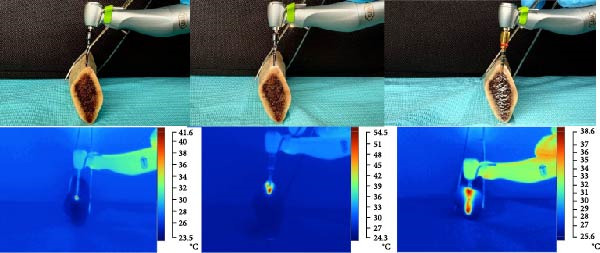
Thermographic‐infrared changes (°C) with different implant systems.

Throughout each of the stages of the surgery, the temperature was monitored with a Fluke TiS55+ thermographic camera (Everett, Washington, USA) at 30 cm from the drilling area. Thermal readings were taken every 10 s to capture the real‐time thermal changes occurring in a 1 cm^2^ perimeter around the milling site. The Fluke camera was precalibrated to ensure the accuracy of data collection. The continuous measurements enabled a careful analysis of the effects of different implant systems and motor types on heat generation during the milling process. The findings were reflected in the analysis of thermal variations, which formed the basis for optimizing surgical procedures with a view to minimizing the risk of thermal damage to the bone tissue (Figure 2).

### 2.8. Analysis Plan

Stata 17.0 software was used. Measures of central tendency and dispersion were calculated as mean and standard deviation for numerical variables such as the temperature of the final drill (third drill °C), the baseline temperature (baseline °C), the temperature with the pilot drill (pilot drill °C), and the temperature with the second drill (second drill °C). Then, the Shapiro–Wilk test and box‐plot plots were applied to evaluate the normality and the homogeneity of variances test. For inferential statistics, the analysis of variance tests (ANOVA) were used. Finally, a multiple linear regression analysis was performed to predict the value of the dependent variable (final milling temperature) based on the value of the other independent variables: basal temperature, temperature with the pilot drill, temperature with the second drill, implant system, and motor type. The regression coefficients were interpreted to identify the magnitude and direction of the relationship between the independent variables and the final drill temperature.

## 3. Results

**Figure 3:**
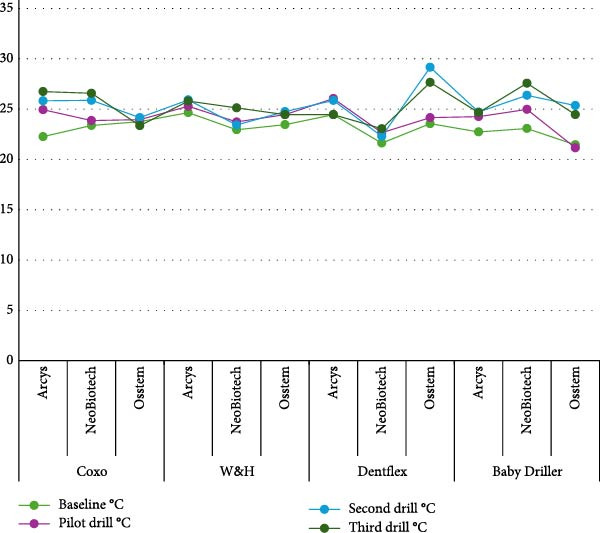
Impact of the diameter, motors, and dental implant system on infrared thermographic variations.

With the Coxo motor, the temperatures reached with the Arcsys system were 24.9°C with the pilot drill, 25.8°C with the second drill, and 26.7°C with the third drill, while with the Osstem system, the temperatures were 23.9, 24.1, and 23.3°C, respectively. With the same Coxo motor, the temperatures achieved with the NeoBiotech system were 23.8, 25.8, and 26.5°C, and with the Osstem system, 23.9, 24.1, and 23.3°C. Statistical analysis by ANOVA revealed significant differences between the systems and the types of strawberries used (*p* = 0.001; Table 2; Figure 3).

**Table 2:** Comparison of motor type, diameter, and dental implant system on thermographic‐infrared changes during surgical osteotomy.

Dental implant motor	System	Baseline (°C) mean ± SD		Pilot drill (°C) mean ± SD		Second drill (°C) mean ± SD		Third drill (°C) mean ± SD		^∗^ *p*
Coxo	Arcys^a^	22.2	0.4	24.9	0.2	25.8	0.4	26.7	0.6	>0.05
	NeoBiotech^b^	23.3	0.1	23.8	0.1	25.8	0.1	26.5	0.1	
	Osstem^c^	23.7	0.1	23.9	0.1	24.1	0.1	23.3	0.1	
	^∗∗^ *p*	0.001		0.001		0.001		0.001		—
W&H	Arcys^a^	24.6	0.3	25.2	0.3	25.9	0.2	25.8	0.3	>0.05
	NeoBiotech^b^	22.9	0.1	23.7	0.1	23.4	0.9	25.1	0.1	
	Osstem^c^	23.4	0.1	24.4	0.1	24.7	0.1	24.4	0.1	
	^∗∗^ *p*	0.001		0.001		0.001		0.001		—
Dentflex	Arcys^a^	24.4	0.1	26.0	0.1	25.8	0.1	24.4	0.1	>0.05
	NeoBiotech^b^	21.6	0.1	22.6	0.3	22.2	0.1	23.0	0.1	
	Osstem^c^	23.5	0.1	24.1	0.1	29.1	0.1	27.6	0.1	
	^∗∗^ *p*	0.001		0.001		0.001		0.001		—
Baby Driller	Arcys^a^	22.7	0.1	24.2	0.1	24.7	0.1	24.6	0.1	>0.05
	NeoBiotech^b^	23.0	0.1	24.9	0.1	26.3	0.1	27.5	0.1	
	Osstem^c^	21.4	0.1	21.1	0.1	25.3	0.1	24.4	0.1	
	^∗∗^ *p*	0.001		0.001		0.001		0.001		—
	^∗∗∗^ *p*	0.001		—	0.006	0.075		0.424		—

^∗^ *p* ^∗^Shapiro–Wilk normality test.

^∗∗^ *p*One‐way ANOVA test between systems (Arcsys, NeoBiotech, and Osstem) by type of drill.

^∗∗∗^ *p*One‐way ANOVA test between systems (Arcsys, NeoBiotech, and Osstem) by type of motor.

^a^Arcsys: Titanium nitride coated drills, the drilling sequence was pilot drill Ø2.4 mm, the next drill Ø 2.9 mm, and 3.4 mm.

^b^NeoBiotech: The initial drill is called Lindermann Ø2 mm, Ø2.2 mm, and Ø3.0 mm.

^c^Osstem: The initial drill is called LanceDrill, Ø2.2 mm and Ø3.0 mm.

With the W&H motor, the temperatures reached with the Arcsys system were 25.2°C with the pilot drill, 25.9°C with the second drill, and 25.8°C with the third drill, while with the Osstem system, the temperatures were 24.4, 24.7, and 24.45°C, respectively. In addition, the temperatures reached with the NeoBiotech system were 23.7, 23.4, and 25.12°C. Statistical analysis using the ANOVA test revealed significant differences between the systems and the types of burs used (*p* = 0.001; Table 2).

With the Dentflex motor, the temperatures reached with the Arcsys system were 26.0°C with the pilot drill, 25.8°C with the second drill, and 24.4°C with the third drill, while with the Osstem system, the temperatures were 24.1, 29.1, and 27.6°C, respectively. On the other hand, the temperatures reached with the NeoBiotech system were 22.6, 22.2, and 23.0°C. Statistical analysis using the ANOVA test revealed significant differences between the systems and the types of drills used (*p* = 0.001; Table 2).

With the Baby Driller motor, the temperatures reached with the Arcsys system were 24.2°C with the pilot drill, 24.7°C with the second drill, and 24.6°C with the third drill, while with the Osstem system, the temperatures were 21.1, 25.3, and 24.4°C, respectively. On the other hand, the temperatures reached with the NeoBiotech system were 24.9, 26.3, and 27.5°C. Statistical analysis using the ANOVA test revealed significant differences between the systems and the types of drills used (*p* = 0.001; Table 2).

With the Arcys implant system, the temperatures reached with the second drill were 25.8°C and with the third drill 26.7°C. With the NeoBiotech system, the temperatures were 25.8°C with the second drill and 26.5°C with the third drill, while with the Osstem system, the temperatures were 24.1 and 23.3°C, respectively. In another data set, with the W&H engine, the temperatures achieved for the Arcsys were 25.9°C with the second bur and 25.8°C with the third bur. With the NeoBiotech system, the temperatures were 23.4 and 25.1°C, and with the Osstem system, the temperatures were 24.7 and 24.4°C. In the third set of data, with the Dentflex engine, the temperatures reached for Arcys were 25.8°C with the second bur and 24.4°C with the third bur. With the NeoBiotech system, the temperatures were 22.2 and 23.05°C, and with the Osstem system, the temperatures were 29.1 and 27.6°C. Finally, in the fourth data set, with the Baby Driller engine, the temperatures reached for the Arcsys were 24.7°C with the second drill and 24.6°C with the third drill. With the NeoBiotech system, the temperatures were 26.3 and 27.5°C, and with the Osstem system, the temperatures were 25.35 and 24.4°C. Statistical analysis using the ANOVA test revealed no significant differences between the systems and the types of burs used (*p* = 0.075 and *p* = 0.424; Table 2).

For each degree that the baseline temperature (baseline°C) increases, the final milling temperature (third drill°C) decreases by 0.67°C (95% CI: −0.93 to −0.41). This is statistically significant. For each degree that the temperature increases with the pilot drill°C, the temperature of the final drill (third drill°C) increases by 0.37°C (95% CI: 0.11–0.62). This is statistically significant. For every degree that the temperature increases with the second drill °C, the temperature of the final drill (third drill°C) increases by 0.80°C (95% CI: 0.70–0.90). This is statistically significant. The implant system had no significant impact on the final drill temperature (third drill°C) (95% CI −0.43 to 0.68). Finally, for each degree increase in temperature with motor type, the final drill temperature (third drill°C) decreased by 0.22°C (95% CI −0.36 to −0.08), which is statistically significant. These results indicate that the basal, pilot drill, and second drill temperatures are important factors in predicting the final drill temperature of the third drill. While the implant system did not have a significant effect, the motor type showed a significant but inverse relationship (Table 3).

**Table 3:** Linear regression model of final drilling.

Variables	Third drill (°C)
	*β* crude
Baseline °C	−0.67°C (IC95%: −0.93 to −0.41)
Pilot drill °C	0.37°C (IC95%: 0.11 to 0.62)
Second drill °C	0.80°C (IC95%: 0.70 to 0.90)
Implant system	−0.18°C (IC95%: −0.43 to 0.68)
Motor type	−0.22°C (IC95%: −0.36 to −0.08)

It was observed that the final milling temperature (third drill°C) varied depending on the implant system and the type of motor used. In general, the Coxo and Dentflex brand motors showed higher temperatures compared to other motors. For example, for the Osstem implant system, the Dentflex motor reached a final temperature of 27.65°C, while the Coxo motor reached a final temperature of 23.35°C. On the other hand, the W&H and Baby Driller motors presented more consistent temperatures and within a narrower range. These results suggest that the choice of motor type and implant system can significantly influence the temperatures achieved during the final drilling, which is crucial for minimizing thermal damage to tissues during surgery (Figure 3).

## 4. Discussion

The results were summarized in a general table, where it was possible to observe the average temperature of each drill belonging to its implant system according to the motor used to perform osteotomy. The highest temperature recorded with the Coxo motor was 26.74°C, using the largest drill of the Arcsys system (3.4 mm), which was also the highest value of this system. Similar maximum temperatures were reached with the W&H motor and Baby Driller, 25.91°C with the second drill of the Arcsys system (2.9 mm), and 27.57°C with the third drill of the NeoBiotech system (3.0 mm). The Dentflex motor achieved the highest overall temperature at 29.15°C using the second bur of the Osstem system (2.2 mm). ANOVA statistical analysis revealed significant differences in all burr types and systems used (*p* = 0.001). However, the same test indicated no significant differences between the motor types and implant systems used.

The success of implant treatment depends on many factors, such as the density of the drilled bone, the osseointegration ensuring anchorage, the necrotic involvement of the bone tissue, and especially the stability of the device [15]. The latter provides crucial information to clinicians because it evaluates the stiffness and strength of the implant, as well as possible complications in fixation to the jaw. It has two divisions: the primary stability takes place when the device is inserted, determined by the bone condition and volume, drilling sequence, and macrostructural design, as well as its own factors such as design and distance between threads, diameter, and total length, among others [15–17]. In addition, higher values are found in bones with densities D1 and D2, compared with densities D3 and D4 [17]. Secondary stability occurs when the bone heals on the implant surface and is mainly influenced by the structure and properties of the implant [16, 17].

The literature establishes a thermal threshold for bone necrosis at values close to 47°C for 1 min; similarly, reducing the time by 30 s but increasing the temperature to 50°C produces the same necrotic effect [18]. To avoid high temperatures, a milling sequence is used, starting with the so‐called pilot drill (~2.00 mm) and progressively increasing the diameter of the drills in the range from 0.2 to 0.7 mm. Just as the drill diameter and drilling sequence influence the temperature control, other factors are taken into account, such as the manufacturing material, rotational speed, irrigation, output torque, and motor type, among others [18, 19].

In a study by Raj et al. [4], a bovine femur was used to perform osteotomy, a COXO Dental Physiodispenser motor, evaluated from two drills of different sizes (2.0 and 2.8 mm), rotation speeds at 1500, 2000, and 2500 rpm, external irrigation (saline solution) at room temperature and 0°C, and forces of 1.2 and 2.4 kgf in manual pressure. The temperature was recorded by infrared thermography. The evaluation according to the diameter and the three speeds showed that a speed of 2000 rpm produced a higher temperature than the others, particularly with the 2.0 mm drill bit. Likewise, the 2.8‐mm drill bit at 1500 rpm emitted less heat. The other evaluations showed that the manual pressure of 2.4 kgf and irrigation at 0°C generated less heat. It is important to mention that in none of the evaluations was the bone necrosis threshold exceeded [4].

Similarly, Soldatos et al. [20] conducted an in vitro study on fresh human tibiae, recording temperature changes during osteotomies performed using Densah drills compared with MIS drills. Three different speeds were used (800, 1000, and 1200 rpm) together with external irrigation. Different sizes of burs were used: 2.3, 4.0, 4.3, and 4.5 mm in the Densah system, while 2.4, 2.8, 3.2, and 4.0 mm were used in the MIS kit. The results indicated that when all the speeds were used in the Densah system, an increase in the bit diameter was associated with a decrease in the temperature. However, in the MIS kit, this trend was not observed at speeds of 800 and 1200 rpm. In any case, in no case was the temperature higher than 47°C observed [20].

In another study, Soldatos et al. [21] evaluated the thermal changes produced by burs of different diameters (1.6, 2.0, 2.3, 2.5, 3.0, 4.0, and 4.3 mm) when performing a clockwise and counterclockwise drilling sequence using a dissected human tibia. For each direction, three speeds (800, 1000, and 1200 rpm) were used. It was found that in the clockwise direction, the temperature did not vary greatly at all speeds, whereas in the counterclockwise direction, at 800 and 1200 rpm, the thermal value increased considerably, while at 1000 rpm, the heat remained stable. The 3.0 and 4.0 mm burs recorded higher temperatures, even exceeding the established threshold [21].

Gabrić et al. [22] compared the thermal changes produced in rat tibiae with an infrared thermographic camera when subjected to different instruments to perform osteotomies. A contact and noncontact Er:YAG laser were used, both belonging to the same brand, each necessarily configured. A piezoelectric device (W&H) and a low‐speed surgical drill at 1200 rpm were used. All surgical procedures included added irrigation. The maximum temperature for the contact laser was 29.9 ± 0.5°C; for the noncontact laser, it increased dramatically to 79.1 ± 4.6°C; for the piezoelectric device, it was 29.1 ± 0.2°C, and the surgical drill reached 27.3 ± 0.4°C. However, when analyzing the different results and performing statistical tests, it was concluded that the Er:YAG laser with the contact mode and the piezoelectric device have greater benefits in osteotomies avoiding overheating [22].

Although this study provides relevant findings, it is important to mention some limitations it presents to carefully interpret the results. For example, only four types of motors were included (Coxo, W&H, Dentflex, and Baby Driller), compared to the large number available on the market. Similarly, three different implant systems were used (Arcsys, NeoBiotech, and Osstem). When compared with other studies, a reduced use of drills of different diameters was observed, decreasing the diversity of results if a greater variety of sizes were used. On the other hand, the decision was made to perform the osteotomies without irrigation with physiological saline solution in order to exclusively evaluate the thermal changes produced by the drills. Finally, considering that this research was carried out on bovine ribs, the results cannot be transferred in their entirety to clinical practice [23, 24], given that there are various factors present in the oral cavity that can influence the thermal changes found.

The thermal values reported here stayed well below the critical value for bone necrosis (44–47° C), which has been defined in the literature. This supports the safety of the motor–implant combinations evaluated in a controlled environment without irrigation. The higher‐than‐average temperature values recorded indicate some of the motor–implant combinations typically sit nearer risk thresholds when subjected to clinical stressors (e.g., long drilling times and reduced irrigation). Clinically, these preliminary findings indicate an advantage for motor systems that in general produce lower output temperatures (e.g., W&H or Baby Driller motor with a NeoBiotech or Arcsys system). Finally, while drill diameter was accounted for, future work should also integrate drill material composition, wear over time, and cutting efficiency, which may significantly alter temperature production and hence long‐term success of the implant [20–25].

The creation of heat based on the motor can be attributed to a variety of mechanical and/or technical considerations. Torque delivery directly influences the resistance applied to the bone, while drilling vibration and cutting ability inform the amount of friction and therefore temperature achieved during osteotomy. Moreover, variations in the motor cooling system help explain differences in heat dissipation between devices. All of these factors could plausibly explain the temperature difference reported in our study [18, 20].

To ensure consistency in the wording describing the milled sequence, this terminology is used in the same manner throughout the manuscript, consistently referring to the first, pilot, second, and last burs. Each sequence was specifically related to the implant systems (Arcsys, NeoBiotech, and Osstem), and this clarification allows the milling steps and links, along with the implant systems studied to be more clearly discussed.

Although this investigation occurred in vitro by using bovine rib specimens, which do not fully represent the anatomical complexity and physiology of the human mandible, this model is still useful to detect and measure thermal changes to be isolated during osteotomy. The use of standardized bone samples meant there was no variability due to different bone types or implant systems. However, we acknowledge future studies are still required to understand the clinical impact of our findings, including in vivo studies and mandible‐specific simulations.

The rationale behind choosing four implant motors (Coxo, W&H, Dentflex, and Baby Driller) and three implant systems (Arcsys, NeoBiotech, and Osstem) was because of their expected availability and consistent usage in the clinical and academic experience prevalent in Latin America/Asia, where this study was contextualized. These systems defined different mechanical configurations, costs, and acceptance in clinic practice that would enable appropriate clinical comparisons of thermal characteristics. While not an exhaustive representation of the available systems, the systems chosen reflect a practical application regionally that is representative of use in the region. Future studies could increase sample size with more systems that represent a wider perspective to increase generalization.

The intentional and methodical choice of performing osteotomies without irrigation was intended to isolate the inherent thermal aspects created by the motors and drill sequencing in a standardized format. This choice enabled the treatment of temperature variations as being caused by mechanical factors only while reducing variability from extraneous factors. Nevertheless, we recognize this design is not reflective of a clinical environment in which irrigation is used on a routine basis to reduce harm from heat‐related injury and bone vitality. Thus, even though these findings provide a meaningful baseline, clinical relevance and translational implications will benefit from future work that simulates irrigation protocol within intraoral conditions.

## 5. Conclusions

Taking into consideration the limitations of the research study, thermographic‐infrared changes were observed in the surgical osteotomy, which were related to the type of motor, diameter, and dental implant system. The dental motors used in this study were the Coxo, W&H, Dentflex, and Baby Driller, and the following implant systems were evaluated: Arcys, NeoBiotech, and Osstem. The results showed significant differences in the maximum temperatures achieved by the drills among dental implant systems in different cases, particularly with the Osstem (Dentflex motor) in what is believed to be the true bone temperature increase with each drill used. The choice of high and low speed of the motor and the implant system may play a role in influencing this temperature which is significant to consider clinically. It is important that clinical practitioners select a motor and implant system that is least likely to create thermal damage thermal to surrounding tissues. Overall, the dental motor and implant system have a multiplicative cascade effect to positively advance surgical outcomes by decreasing risk to patient safety.

## Ethics Statement

This project is part of a line of research within a macro project that obtained a grant and was approved under strict ethical standards. The study was approved by the Ethics Committee of the Faculty of Dentistry of the Universidad Nacional Federico Villarreal with Code No. 032‐02‐2025.

## Disclosure

All authors read and approved the final manuscript.

## Conflicts of Interest

The authors declare no conflicts of interest.

## Author Contributions

Frank Mayta‐Tovalino, Berly Delgado‐Cumpa, Julia Medina, and Fran Espinoza‐Carhuancho searched the database and analysis data. Fran Espinoza‐Carhuancho, Julia Medina, Berly Delgado‐Cumpa, Ivan Calderon, and Daniel Alvitez‐Temoche wrote the main manuscript text and prepared the table. Frank Mayta‐Tovalino, Fran Espinoza‐Carhuancho, Franco Mauricio, Ivan Calderon, and Daniel Alvitez‐Temoche analyzed the data. Frank Mayta‐Tovalino, Fran Espinoza‐Carhuancho, Daniel Alvitez‐Temoche, and Ivan Calderon designed, critically reviewed, and modified the manuscript.

## Funding

The study was partially carried out with some previous instruments from another project of the Vice‐Rectorate of Research of the Universidad Nacional Federico Villarreal, Lima‐Peru, under the grant code RESOLUTION R. No. 3919‐2024‐CU‐UNFV.

## Data Availability

The datasets used and/or analyzed during the current study are available from the corresponding author on reasonable request.
